# Supplementary figures and images for: The N-Terminal Part of the 1A Domain of Desmin Is a Hot Spot Region for Putative Pathogenic DES Mutations Affecting Filament Assembly
Source: Cells. 2022 Dec 2;11(23):3906. doi: 10.3390/cells11233906 (PMC9738904; doi:10.3390/cells11233906)

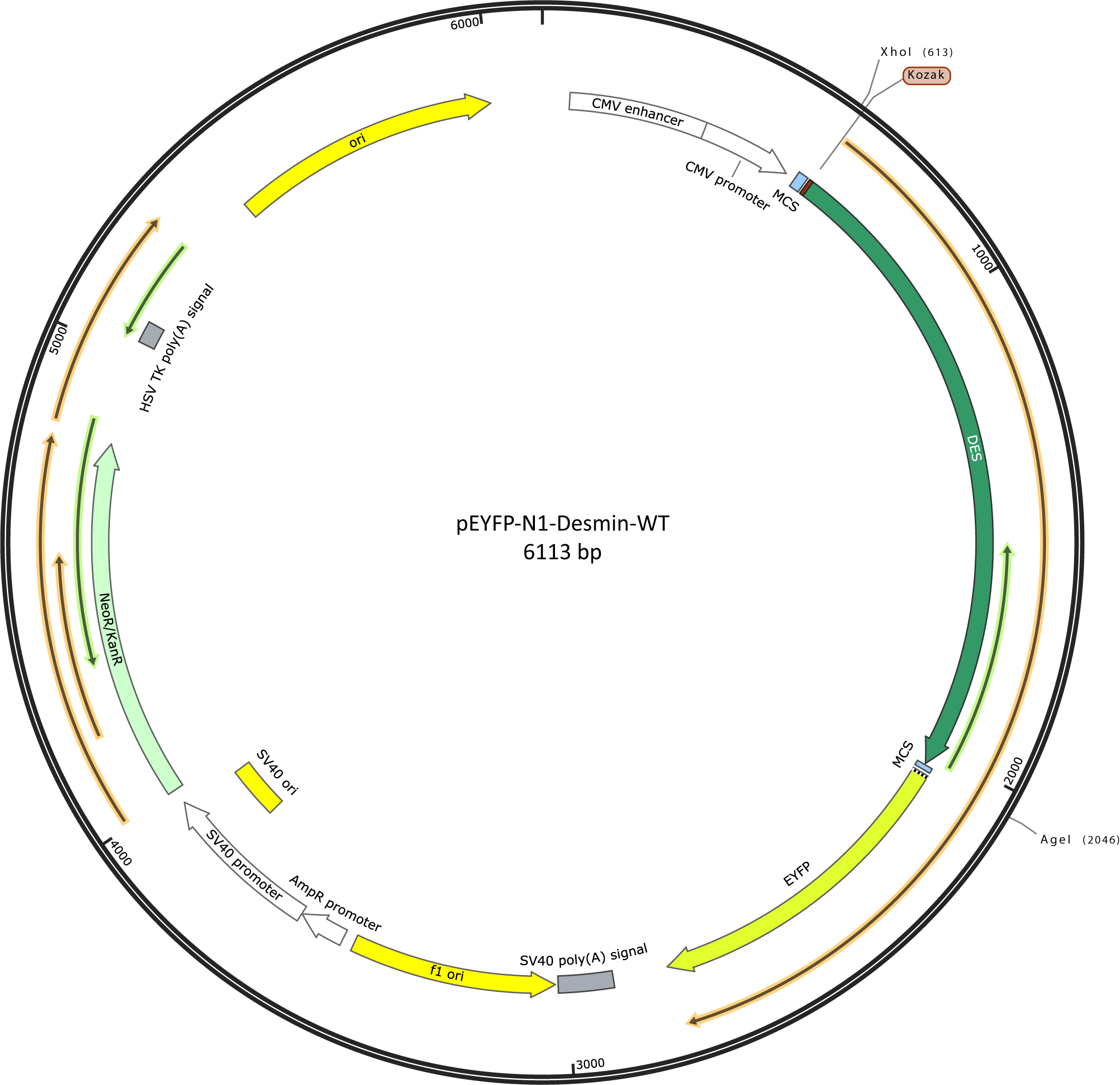

Supplement: Supplementary file 1 [file cells-11-03906-s001.zip › Figure S1.tif]

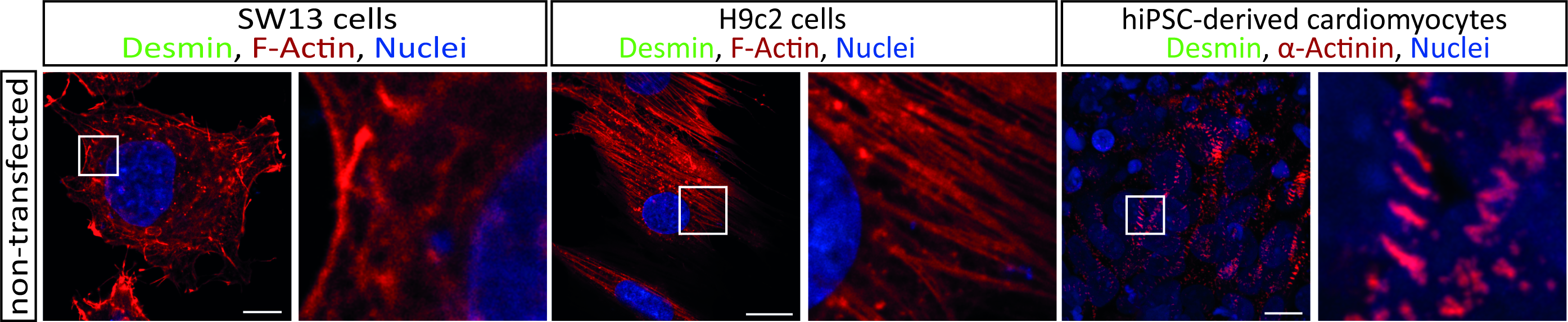

Supplement: Supplementary file 1 [file cells-11-03906-s001.zip › Figure S2.tif]

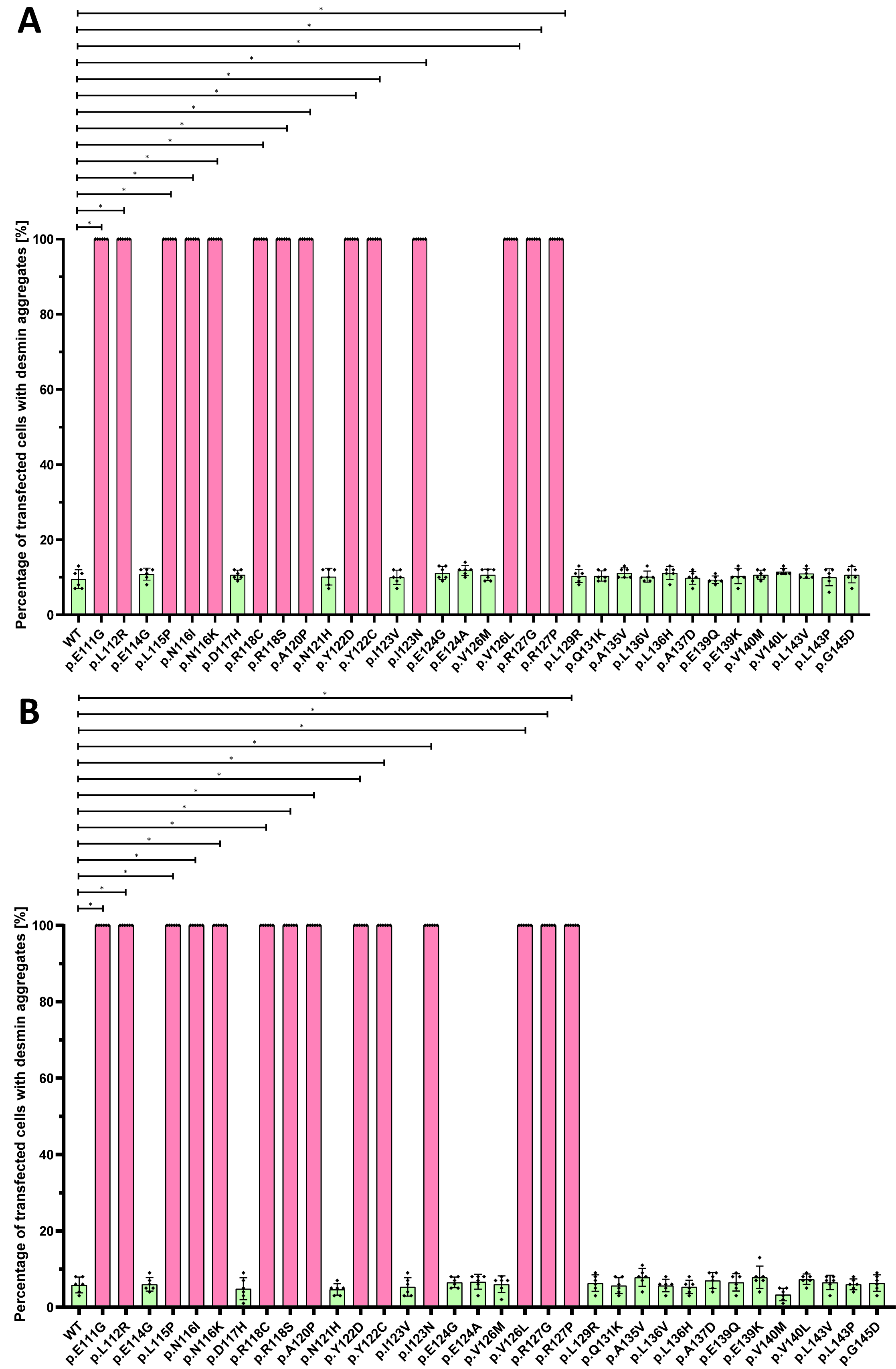

Supplement: Supplementary file 1 [file cells-11-03906-s001.zip › Figure S3.tif]
